# Detecting Newcastle disease virus in combination of RT-PCR with red blood cell absorption

**DOI:** 10.1186/1743-422X-8-202

**Published:** 2011-05-02

**Authors:** Jianzhong Yi, Chengqian Liu

**Affiliations:** 1 Institute of Animal Husbandry Veterinary Sciences, Shanghai Academy of Agricultural Sciences, 2901 Beidi Road, Shanghai, 201106, China

## Abstract

Reverse transcription-polymerase chain reaction (RT-PCR) has limited sensitivity when treating complicated samples, such as feces, waste-water in farms, and nucleic acids, protein rich tissue samples, all the factors may interfere with the sensitivity of PCR test or generate false results. In this study, we developed a sensitive RT-PCR, combination of red blood cell adsorption, for detecting Newcastle disease virus (NDV). One pair of primers which was highly homologous to three NDV pathotypes was designed according to the consensus nucleocapsid protein (NP) gene sequence. To eliminate the interfere of microbes and toxic substances, we concentrated and purified NDV from varied samples utilizing the ability of NDV binding red blood cells (RBCs). The RT-PCR coupled with red blood cell adsorption was much more sensitive in comparison with regular RT-PCR. The approach could also be used to detect other viruses with the property of hemagglutination, such as influenza viruses.

## Introduction

Newcastle disease is a highly contagious disease of birds affecting many domestic and wild avian species. The high susceptibility and mortality often cause epidemics in poultry populations. Transmission occurs by exposure to fecal and other excretions from infected birds, or through contact with contaminated feed, water, equipment and plumage [1]. Hemagglutionation (HA) and ELISA methods have been applied to detect NDVs in chicken embryos and tissues [2]. Although the egg passage test is more sensitive than HA and ELISA [3], it will take several days to obtain results, therefore, a rapid and sensitive method will be helpful to detect virus in varied samples.

RT- PCR has been developed to detect and pathotype Newcastle disease virus (NDV) in clinical samples [4-6]. However, RT-PCR has limited sensitivity in detecting complicated samples, such as feces, tissue samples and contaminated water. Thus, an effective and simple virus concentration and purification method will be great helpful to enhance the detection sensitivity [7,8].

Newcastle disease virus, being a paramyxovirus, causes hemagglutination of chicken red blood cells. There are two viral glycoproteins, HN and F fusion protein, embedded in the viral envelope, HN is responsible for virus attachment to host cell receptors, and the F fusion protein mediates fusion of viral envelope with the host cell membrane, enabling the virus to enter host cells [9]. By virtue of the property of NDV binding to red blood cells (RBCs), we concentrated and purified NDV by a red blood cell adsorption and de-adsorption process, we optimized the conditions of adsorption and de-adsorption to develop an effective method for NDV purification and detection. In this study, one pair of primers was designed according to the relevant sequence of the nucleocapsid protein (NP) gene of NDV acquired from Genbank (accession number FJ766531). We applied the reverse transcription (RT)- polymerase chain reaction (PCR), coupled with RBC adsorption to detect NDV in varied samples. The results of this study demonstrated that the described RBC adsorption RT-PCR assay has the potential to be used for the rapid and sensitive detection of NDV isolates in a variety of samples, and could be used to detect other viruses with the property of hemagglutination.

## Materials and methods

### Virus strains and virus propagation

The La Sota strain came from the commercial vaccine strain. The F48E9 strain was obtained from China Institute of Veterinary Drug Control. The N79 strain was preserved by our laboratory. All the strains were amplified in ten-day-old embryonated SPF eggs

### Preparation of red blood cells

Venous blood (0.5 ml) was collected from 2 month-old healthy chickens with sodium citrate anticoagulant, centrifuged at 750× g for 5 min, the serum and buffy coat were aspirated, the remaining erythrocytes were washed with phosphate buffered saline (PBS) three times by spinning at 750× g for 5 min, then the cells were suspended in 200 μl PBS.

### Efficiency of red blood cells Adsorption

The allantoic fluid (1.0 ml 5 × 10^9 ^EID_50 _) was mixed with 50 μl washed red blood cells, and incubated with rotation (150 rpm) at room temperature for 20 min, After adsorption treatment, The cells were then collected by spinning at 750× g for 5 min. The virus titer in supernatant was tested by the EID assay.

### Optimization of the conditions releasing NDVs from RBC

After NDVs adsorption, the collected cells were suspended with 1 ml saline containing different concentrations of EDTA and β-mercaptoethnol for release of NDV from RBC at 37°C, then spin at 1000χg for 5 min. The supernatant was collected and tested by the EID assay.

### RNA Extraction and RT -PCR

After NDVs de-adsorption, the supernatant was used to extract the NDV genome RNA by Trizol reagent (Invitrogen, USA). One pair of specific primers was designed according to the relevant sequence of the NP gene (FJ766531) in Genebank. The primers were synthesized by Shanghai Generay Biotech Co., Ltd. NP-F1: 5'-TAGAAGGTGTGAACCTCGAG -3', NP-R1: 5'-CTTATGTATGAGTCTACATCC-3',

cDNA was synthesized using 2 μl of the total RNA, 4 μl of 5 × reverse transcriptase buffer, 1 μl of 10 mM dNTP, 1 μl of NP-F1(10 μM), 1 μl of RNase inhibitor (20 U/μl), 1 μl of AMV reverse transcriptase (5 U/μl), and 3 μl double-distilled H_2_O in a total volume of 20 μl for 1 h at 42°C.

A 578-bp fragment of the NP gene was amplified in 25 μl of the reaction mixture containing 2 μl of cDNA, 2.5 μl of 10× PCR buffer, 2 μl of MgSO_4 _(25 mM), 1 μl of dNTP, 1 μl of NP-F1 primer and NP-R1 primer (10 μM), l5.25 μl of double-distilled H_2_O and 0.25 μl of TaKaRa Taq polymerase (5 U/μl). The PCR condition was as follows: 1 cycle at 94°C for 5 min; followed by 35 cycles of 30s at 94°C, 35s at 55°C, 60s at 72°C. The PCR ended with a final elongation for 10 min at 72°C.

### Determination of sensitivity of the RBC coupled RT-PCR

To estimate the sensitivity of the RBC coupled RT-PCR in comparison with regular RT-PCR, Serial 10-fold dilutions of the 1 × 10^9 ^EID_50 _NDVs allantoic fluid were made in PBS buffer. RNA was extracted from 1 ml aliquots of each dilution by red blood cell adsorption approach and regular approach, respectively. The RNA extracts were simultaneously analyzed in parallel by RT- PCR.

### Detection of NDV in fecal samples

The feces (0.2 g) collected from F48E9 infected chickens, and diluted the feces by 1000 and 10000 fold, then centrifuged at 15,000× g for 10 min two times, the supernatant was incubated with 50 μl washed RBC to adsorb NDV particles with rotation (150 rpm) at room temperature for 30 min, the cells were collected by spinning at 750× g for 5 min. After de-adsorption of the NDVs from cells, the supernatant was tested for the presence of NDV with RT- PCR.

### Detection of NDV in different tissue samples by the RBC coupled RT-PCR

To verify whether the approach was applicable to detect complicated samples, liver, kidney, and lung samples were collected from N79 infected chickens and homogenized in 1 ml PBS, then centrifuged at 15,000× g for 10 min two times. After adsorption and de-adsorption process, the samples were tested in parallel by RT- PCR.

### Detection of NDV in infected chickens with different NDV strains

60 4-week-old SPF White Leghorn chickens were hatched and reared in isolation, 15 chickens per group were inoculated with 2 × 10^6 ^EID50 of the following strains of NDV: La Sota, F48E9 and N79, and kept in isolation, four days post inoculation, feces were collected, spleen, brain, and kidneys were also aseptically collected. All the samples stored separately at 70°C until further analysis. Homogenized fecal and tissue samples (0.2 g) from infected chickens were suspended in 1 ml PBS, then centrifuged at 15,000× g for 10 min two times, After adsorption and de-adsorption of the NDVs from red blood cells, the supernatant samples were tested in parallel for the presence of NDV with RT- PCR.

## Results

### Efficiency of red blood cells adsorption and de-adsorption

After treating 1.0 ml allantoic fluid (5 × 10^9 ^EID_50 _) with 50 μl washed red blood cells, almost no viruses could be detected in the supernatant by HA experiment, most of NDVs attached to the surface of red blood cells. After NDVs adsorption, we optimized the de-adsorption conditions by suspending the collected cells in saline with different concentrations of EDTA and β-mercaptoethnol, the results showed 5 mM EDTA and 10 mM β-mercaptoethnol could effectively release NDVs from red blood cell at 37°C, while high concentrations of EDTA and β-mercaptoethnol will lead to hemolysis and low virus titer in the supernatant (Table 1).

**Table 1:** Optimization of the conditions releasing NDVs from RBC 1

β-mercaptoethnol	EDTA				
	
	1.25 mM	2.5 mM	5 mM	10 mM	20 mM
1.25 mM	2.9 × 10^9^	3.4 × 10^9^	4.0 × 10^9^	4.1 × 10^9^	3.9 × 10^9^

2.5 mM	3.1 × 10^9^	3.3 × 10^9^	4.1 × 10^9^	4.2 × 10^9^	3.9 × 10^9^

5 mM	3.5 × 10^9^	3.8 × 10^9^	4.3 × 10^9^	4.2 × 10^9^	3.8 × 10^9^

10 mM	3.7 × 10^9^	4.2 × 10^9^	4.4 × 10^9^	3.9 × 10^9^	3.7 × 10^9^

20 mM	3.6 × 10^9^	4.0 × 10^9^	4.2 × 10^9^	3.6 × 10^9^	3.4 × 10^9^

### Sensitivity of the red blood cell coupled RT- PCR

**Figure 1:**
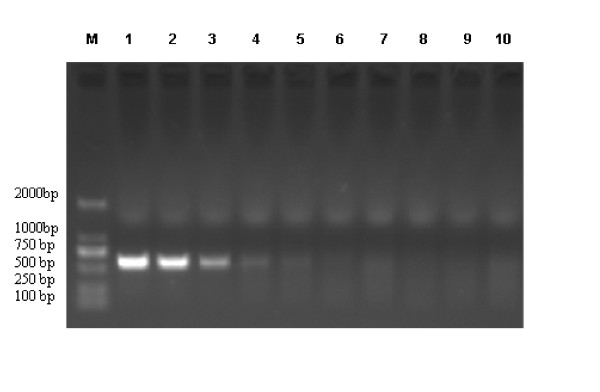
**Assay of the sensitivity of the red blood cell adsorption-coupled RT- PCR in comparison with regular RT-PCR**. To test the sensitivity of the RBC coupled RT-PCR, Serial 10-fold dilutions of the 1 × 10^9 ^EID_50 _NDVs allantoic fluid were made in PBS buffer. RNA was extracted after the red blood cell adsorption and de-adsorption process, with regular approach as control, RNA extracts were simultaneously analyzed in parallel by RT- PCR in comparison with regular RT-PCR approach. Lane 1, Positive control. **Red blood cell adsorption-coupled RT- PCR: **lane 2, 1: 10^5 ^dilution of NDVs allantoic fluid (1 × 10^9 ^EID_50 _); lane 3, 1: 10^6 ^dilution of NDVs allantoic fluid (1 × 10^9 ^EID_50 _); lane 4, 1: 10^7 ^dilution of NDVs allantoic fluid (1 × 10^9 ^EID_50 _); lane 5, 1: 10^8 ^dilution of NDVs allantoic fluid (1 × 10^9 ^EID_50 _).**Regular RT-PCR: **lane 6, 1: 10^5 ^dilution of NDVs allantoic fluid (1 × 10^9 ^EID_50 _); lane 7, 1: 10^6 ^dilution of NDVs allantoic fluid (1 × 10^9 ^EID_50 _); lane 8, 1: 10^7 ^dilution of NDVs allantoic fluid (1 × 10^9 ^EID_50 _); lane 9, 1: 10^8 ^dilution of NDVs allantoic fluid (1 × 10^9 ^EID_50 _). Lane 10, negative control.

To assess the sensitivity of red blood cell adsorption-coupled RT-PCR, we serially diluted the NDV allantoic fluid. The limit of NDV detection was in the range of 10^7 ^fold diluted samples (100 EID_50_) (Figure 1), which is much more sensitive than the regular RT-PCR on agarose electrophoresis detection.

### Sensitivity of NDV detection in fecal samples

**Figure 2:**
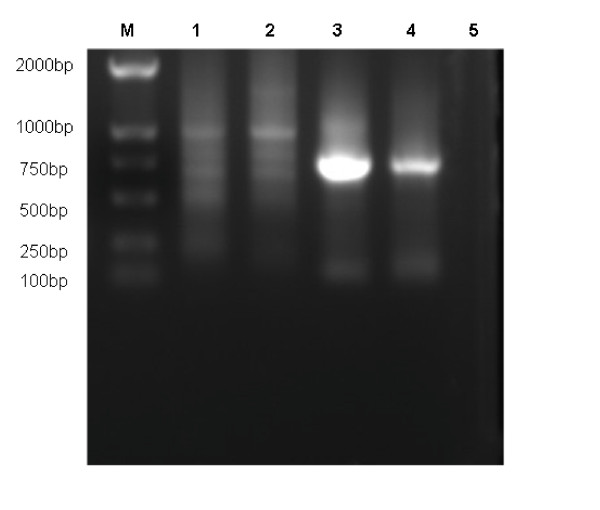
**The sensitivity of detection of NDV in fecal samples**. The feces (0.2 g) collected from F48E9 infected chickens, diluted in serial 10-fold, was detected by red blood cell adsorption-coupled RT- PCR in comparison with regular RT-PCR approach. **Regular RT-PCR: **lane 1, 1: 10^3 ^dilution of feces in PBS, lane 2, 1: 10^4 ^dilution of feces in PBS. **Red blood cell adsorption-coupled RT- PCR: **lane 3, 1: 10^3 ^dilution of feces in PBS, lane 4, 1: 10^4 ^dilution of feces in PBS. Lane 5, negative control.

The efficiency of the established RT-PCR assay was assessed in diluted fecal samples from infected chickens. After gel electrophoresis, a 578 bp band was visible until the 10^4 ^fold dilution (Figure 2), therefore, RT-PCR, coupled with red blood cell adsorption, is much more sensitive than the regular RT-PCR in detecting NDVs feces.

### Detection of NDV in different tissue samples

**Figure 3:**
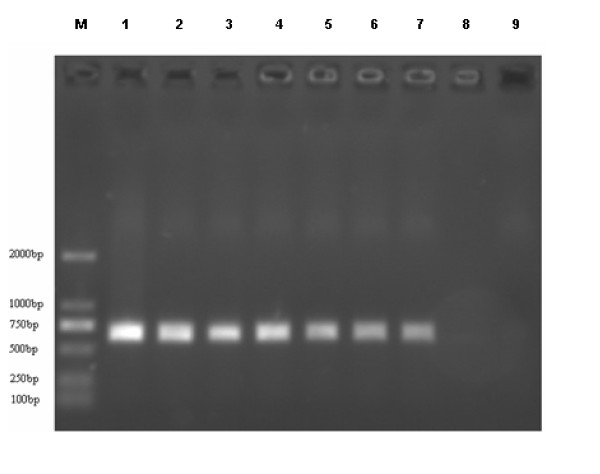
**Detection of NDV in tissue samples**. Liver, kidney, and lung samples from N79 infected chickens were homogenized in 1 ml PBS, after adsorption and de-adsorption process, the samples were tested in parallel by RT- PCR. Lane 1, Positive control. Lane 2, liver sample 1, lane 3, liver sample 2. Lane 4, kidney sample 1, lane 5, kidney sample 2. Lane 6, lung sample 1, lane 7, lung sample 2. Lane 8, Negative control. Lane 9, PCR blank.

To assess whether the approach is applicable to tissue samples, the liver, kidney, and lung samples from chickens infected with N79 NDVs were screened by RT-PCR after the adsorption and de-adsorption process (Figure 3). The positive RT-PCR in each samples demonstrated that the approach was applicable to detect NDVs in different kind of samples.

### Detection of NDV in the fecal and tissue samples infected with different strains

To test whether the approach is effective in detecting different NDV strains, SPF chickens were inoculated with La Sota, F48E9, and N79 strain, respectively. After 4 days, the feces and tissue samples were collected and detected by red blood cell coupled RT-PCR in comparison with regular RT-PCR. RT-PCR results of samples from the inoculated animals are summarized in Table 2. The results showed the new approach could detect different NDV strain in all the varied samples, while regular RT-PCR only detected part of the samples. This demonstrates that red blood cell coupled RT-PCR is a rapid and efficient method for the detection of NDV in varied strains as well as varied samples.

**Table 2:** Comparison of RBC coupled-RT-PCR and regular PCR in the detection of NDVs in feces and tissues of infected chickens

	NDV Virus											
	**La Sota**			**F48E9**			**N79**			**Negative control**		
				
	**feces**	**kidney**	**liver**	**feces**	**kidney**	**liver**	**feces**	**kidney**	**liver**	**feces**	**kidney**	**liver**

RBC												
RT-PCR	15+	15+	15+	15+	15+	15+	15+	15+	15+	15-	15-	15-

Regular												
RT-PCR	7+	9+	8+	8+	12+	11+	7+	10+	11+	15-	15-	15-

## Discussion

NDV has a variety of strains that differ widely in virulence, from causing an asymptomatic infection to lethal disease. Although vaccination programs have provided significant protection against NDV outbreaks, infections by ND viruses have been reported frequently around the world in recent years. Correct and fast diagnosis of NDV will surely help in effective controlling the disease. Unfortunately, rapid detection of NDV at the onset of the disease has always been hampered by the lack of a sensitive and fast detection method. Current diagnosis of NDV infection by conventional virus isolation and serological tests, such as haemagglutination inhibition (HI) and serum neutralization, are either time-consuming or lack the required sensitivity [10-12].

Feces from NDV infected birds play an important role within the spread of NDV, however, the bacterial contamination and toxic substances in feces limit NDV detection by virus isolation in embryonated eggs, therefore, RT-PCR is a valuable alternative. It has been reported that some organic substances present in feces and tissue samples would inhibit the activity of reverse polymerase and Taq polymerase [13]. Furthermore, the large amount of celluar RNA in tissue samples will interfere with the RT-PCR reaction, usually leading to false positive and negative results, so the property of the samples is critical for obtaining RNA of good quality, however, the conventional RNA extraction methods couldn't get rid of these problems, rather sometimes facilitate contamination and loss of virus particles during sample preparation, especially due with low viral load samples [14].

In this study, we concentrated and purified NDVs by a red blood cell adsorption and release process depending on the ability of NDV binding to RBC, we could thus get rid of the contaminant substances and cellular RNA. This approach has been proved to be much more sensitive than the regular RT-PCR in treating varied samples and different NDV strains. Therefore, combining the RT- PCR and red blood cell adsorption is useful when the amount of NDV particles is too low to be detected during the onset of an outbreak, or possibly when it is necessary to trace the origin of the different viruses. In recent years, duplex polymerase chain reaction, real time PCR, and molecular probes have been developed to detect NDVs in different samples with high sensitivity [15,16], when combination of these new PCR methods with the red blood cell purification procedure will greatly enhance the detection sensitivity and decrease false results.

## Competing interests

The authors declare that they have no competing interests.

## Authors' contributions

JZ participated in the design of the study, performed RT-PCR assay, data analysis, and drafted the manuscript. CQ participated in the optimizing NDV- red blood cell adsorption and release process. All authors read and approved the final manuscript.
